# Calpain-1 Expression in Triple-Negative Breast Cancer: A Potential Prognostic Factor Independent of the Proliferative/Apoptotic Index

**DOI:** 10.1155/2017/9290425

**Published:** 2017-04-27

**Authors:** Shadia M. Al-Bahlani, Ruqaya M. Al-Rashdi, Shiyam Kumar, Shadia S. Al-Sinawi, Maiya A. Al-Bahri, Asem A. Shalaby

**Affiliations:** ^1^Department of Allied Health Sciences, College of Medicine and Health Sciences, Sultan Qaboos University, Muscat, Oman; ^2^Department of Pathology, Sultan Qaboos University Hospital, Sultan Qaboos University, Muscat, Oman; ^3^Oncology Unit, Department of Medicine, Sultan Qaboos University Hospital, Sultan Qaboos University, Muscat, Oman

## Abstract

Triple-negative breast cancer (TNBC) is an aggressive type of breast cancer in which calpain system plays an important role in its cellular processes including apoptosis and proliferation. Although such roles have been assessed in tumor pathogenesis, the correlation of its expression to the proliferating/apoptotic index has not been studied yet. Immunohistochemical staining of calpain-1 was performed on paraffin-embedded tissues to correlate its expression with clinicopathological variables and outcome. The proliferation activity was determined by calculating the percentage of cells expressing the Ki-67 antigen. The apoptotic index was assessed morphologically and biochemically using Haematoxylin & Eosin method and Terminal deoxynucleotidyl transferase-mediated dUTP nick end labeling assay, respectively. Calpain-1 was significantly expressed in TNBC tissues varying from low to high with a significant correlation to lymph node status but not with the other clinicopathological variables, suggesting its role as a prognostic factor. In addition, a positive correlation was found between both apoptotic counts assays (*P* < 0.001, *r* = 0.547) as well as with proliferation (*P* = 0.045). Calpain-1 expression had no significant correlation with either proliferation (*P* = 0.29) or apoptotic indices (*P* = 0.071 and *P* = 0.100). Determining calpain-1 expression may provide relevant prognostic value for TNBC cancer patients.

## 1. Introduction

Breast cancer is a heterogeneous disease with a marked diversity in its morphological appearances and behaviors that influence its progression and thus therapeutic resistance [1]. Triple-negative breast cancer (TNBC) is an aggressive form of breast cancer that lacks estrogen (ER), progesterone (PR), and the HER-2 receptors, the most known receptors that affect breast tissue growth. Therefore, hormonal- and targeted-therapies are considered to be ineffective whereas platinum-based drugs can be an effective option [2–5].

Calpains are calcium (Ca^2+^) dependent, nonlysosomal cysteine proteases that are found in all mammalian cells [6, 7]. The calpain family members can be classified into two major groups according to their tissue distribution: the tissue-specific and the ubiquitously expressed [6]. Calpain-1 (*μ*-calpain) and calpain-2 (m-calpain) are the most common isoforms that are ubiquitously expressed [7–9]. They exist in the cytosol and the cellular membrane as inactive proenzymes but they are activated by an intracellular increase in Ca^2+^ concentration. Both *μ*-calpain and m-calpain are called so, based on their Ca^2+^ concentration requirement for in vitro activation, 2–80 *μ*M and 0.2–0.8 mM of Ca^2+^ for calpain-1 and calpain-2, respectively [6]. The calpain system has been implicated in several pathophysiological phenomena [10, 11]. Calpain-1 plays a crucial role in different processes crucial for cancer biology activity by involving in many important cellular processes cell proliferation and apoptosis [7–9, 11–13].

Whether the cell enters proliferation or apoptotic pathway is determined by inducers and repressors that control its activity. Key features for tumors to grow are continued proliferation and the suppression of apoptosis by oncogenic mutations. Conversely, compelling evidence has shown that some oncogenic mutations can also promote apoptosis. This enacts from the notion that overexpression of some oncoproteins can provoke counteracting responses. Alternatively, some cancer cells may adapt to high levels of oncoproteins by disabling their apoptotic machinery [14]. With these phenomena, it is hypothesized that proliferation and apoptotic processes may be tightly coupled (Liu et. al., 2001). Proliferation is widely estimated by the immunohistochemical assessment of the nuclear antigen Ki-67. Apoptotic cells are frequently quantified by counting morphologically positive cells on an H&E stain or evaluating the number of TUNEL-positive cells per 1000–2000 cells. Such analyses of cell proliferation and apoptosis provide a strong basis for tumor doubling time, act as independent prognostic factors, and are used to guide treatment protocols in clinical practice [15]. Despite its importance, very few studies have addressed this concept in cancer types. For example, a correlation between the apoptotic and survival rates in patients with colorectal carcinoma demonstrated that a higher apoptotic index is associated with more aggressive tumors and a poorer prognosis [15, 16]. Another study examined the prognostic significance of apoptotic factors in gastric cancer in which they suggested that simultaneous evaluation of the proapoptotic p53 and the antiapoptotic Bcl-2 in patients with gastric adenocarcinoma may have prognostic significance [17].

The aim of the current study is to assess the proliferating/apoptotic index in triple-negative breast cancer (TNBC) tissue and correlate it to calpain-1 expression. Here we demonstrate that TNBC tissues significantly express calpain-1, varying from low to high with a significant correlation with the lymph node status, suggesting its role as a prognostic factor. Interestingly, such expression did not correlate with either proliferation or other clinicopathological variables. In addition, both approaches for apoptotic counts reveal a significant association, yet calpain-1 expression did not correlate with apoptosis. The lack of correlation between calpain-1 expression and the proliferative/apoptotic index suggests that calpain-1 is an independent prognostic factor. Such correlation will provide new insights to a better understanding of breast cancer behavior through discovering new potential biomarkers and thus new therapies.

## 2. Materials and Methods

### 2.1. Reagents

Immunohistochemistry (IHC) was performed using ultraView DAB Detection Kit on a BenchMark ULTRA automated staining system from Ventana Medical Systems (Tucson, Arizona). Mouse anti-calpain-1 (1 : 1000) antibody was purchased from Santa Cruz Biotechnology (Dallas, Texas, clone 15C10) while mouse anti-ki-67 (1 : 50) antibody was purchased from Dako (Glostrup, Denmark, clone MIB-1). Slides were mounted using Tissue-Tek Film Automated Coverslipper from Sakura Finetek Japan (Tokyo, Japan). TUNEL assay was performed using DeadEnd Colorimetric TUNEL kit which is purchased from Promega Corp (Madison, Wisconsin). Xylene was purchased from Fisher Scientific (England, UK). Ethanol was purchased from VWR International (Dublin, Ireland). Paraformaldehyde and Phosphate Buffered Saline were purchased from Micro Essential Laboratory (Brooklyn, New York). H&E Staining was performed by Tissue-Tek Prisma machine from Sakura Finetek Japan (Tokyo, Japan). Acid alcohol was purchased from VWR International (Dublin, Ireland). Ammonia was purchased from Applichem (Darmstadt, Germany). Eosin was purchased from Riedel-de Haën (Hanover, Germany). Slides were screened by light microscope manufactured by Olympus Corporation (Tokyo, Japan).

### 2.2. Patient Samples

The present study was approved by the Ethics Committee of College of Medicine & Health Sciences, Sultan Qaboos University (MREC#670). A total of 55 patients were diagnosed with TNBC during the study period (April 2008 to February 2016) and paraffin-embedded tissues were tested for calpain-1 protein expression. All patients were females with a median age of 47 years (19–74). Most of them were premenopausal with no family history of breast cancer. The majority of patients (67.3%) had high body mass index and half of them (50.9%) had stage III or IV disease at the time of diagnosis. Approximately, 76.4% of them had surgery as a primary treatment. Almost half of the patients (*n* = 26, 47.3%) received neoadjuvant treatment and 5 (19.2%) achieved complete pathological response. Anthracyclines and taxanes were the most commonly used chemotherapeutic agents as front line treatment. Breast cancer related overall survival (OS) was defined as the time interval (in months) from the date of diagnosis until death from breast cancer. Similarly, recurrence-free survival (RFS) was defined as the time interval (in months) between the start of primary treatment and date of cancer relapse.

### 2.3. Immunohistochemistry

Immunohistochemistry (IHC) was performed to measure the expression of calpain-1 and ki-67 (the cellular marker for proliferation), using the automated staining system as described earlier [18]. Briefly, the tissue sections were baked at 72°C for 4 minutes, deparaffinized with EZ Prep at 72°C, and heat pretreated in Cell Conditioning 1 using “standard cell conditioning” for antigen retrieval at 95°C for 36 minutes. Endogenous peroxidase was then blocked by hydrogen peroxide for 4 minutes and then slides were incubated with mouse anti-calpain-1 primary antibody for 32 min at 37°C. They were then treated with a copper-enhanced DAB reaction. The slides were counterstained with Hematoxylin II for 8 min and Bluing Reagent for 4 min and then cover-slipped. Positive control (non-triple-negative breast cancer tissue) was treated the same way as the sample tissues. The negative control (non-TNBC tissue) was performed without the addition of calpain-1 antibody. Finally, the stained tissue was screened under light microscope where the staining intensity of calpain-1 in tumor cells was assessed as none (0), weak (1), medium (2), and strong (3) as described previously [19]. Ki-67 expression was estimated as the percentage of tumor cells stained by the antibody per field using ×40 objective as described earlier [19].

### 2.4. Haematoxylin and Eosin (H&E) Staining

The H&E staining was performed by Tissue-Tek Prisma machine. Sections were deparaffinized and rehydrated as above and then were washed for 3 minutes, followed by immersing them in Hematoxylin for 10 minutes. Sections were then washed twice for 1 minute and 30 seconds, respectively, and then were differentiated with 1% acid alcohol for 5 seconds, washed for 45 seconds, blued with ammonia for 45 seconds, washed for 45 seconds, stained with 1% Eosin for 7 minutes, and then washed for 35 seconds and dehydrated with 3 changes of absolute alcohol (15 seconds each) and 3 changes of xylene (for 1, 3, and 3 minutes, resp.). Finally, the tissue sections were cover-slipped. Apoptotic cells were counted per 100 invasive tumor cells, using ×40 objective as previously described [20].

### 2.5. TUNEL Assay

TUNEL assay was used for specific labeling of nuclear DNA fragmentation according to the manufacturer's instructions. Briefly, sections were deparaffinized by immersing them in xylene and rehydrated in a series of graded concentrations of ethanol, followed by fixing them in 4% paraformaldehyde for 15 minutes. They were incubated with 20 *μ*g/mL of proteinase K for 20 minutes at room temperature, and then sections were rinsed with PBS and kept in the equilibration buffer for 8 minutes at room temperature. They were incubated with 100 *μ*L of working rTdT enzyme in a humid atmosphere for 1 hour at 37°C. The reaction was terminated by immersing the slides in 2x SSC solution for 15 minutes. The sections were then immersed in 0.3% hydrogen peroxide for 5 minutes at room temperature to block endogenous peroxidase and then were rinsed with PBS and incubated with 100 *μ*L of streptavidin HRP for 30 minutes at room temperature. Then they were rinsed with PBS, and the sections were stained with DAB. Positive control (human colon mucosa tissue) was treated the same way as the sample tissues. The negative control (human colon mucosa tissue) was performed by incubating the sections with rTdT reaction mix without the rTdT enzyme. An experienced observer reviewed all the slides by using an Olympus BX51 microscope. In TUNEL assay, biotinylated nucleotide is incorporated at the 3′-OH DNA ends using the Recombinant Terminal Deoxynucleotidyl Transferase (rTdT) enzyme. Streptavidin horseradish peroxidase conjugate is then bound to the biotinylated nucleotides, which are then detected using the peroxidase substrate, hydrogen peroxide, and the stable chromogen, diaminobenzidine (DAB), resulting in dark brown staining of the apoptotic nuclei. Human colon mucosa tissue was used as a positive control slide which was processed along with the tested cases (Liu et al., 2001). The number of stained cells that exhibited apoptotic-like morphology was assessed by counting the apoptotic cells per 100 cells in a randomly chosen field using ×40 objective.

### 2.6. Statistical Analysis

The distribution of data was assessed using the Kolmogorov–Smirnov test. Pearson *χ*^2^ test of association was used for correlation between categorized protein expression and clinicopathological variables and for correlation between calpain-1 expression and proliferation. The correlation between calpain-1 expression and apoptotic TUNEL assay and the correlation between proliferation and apoptosis were assessed using Kruskal-Wallis test. The correlation between calpain-1 expression and morphological apoptotic H&E assay was assessed using one-way ANOVA test. Morphological apoptotic counts were compared with apoptosis data derived from the apoptotic TUNEL assay using Spearman's rho correlation test. Recurrence-free survival (RFS) was calculated from the date of diagnosis to the date of documented recurrence and the overall survival (OS) was calculated from the date of diagnosis to the date of death or censored at the last date of follow-up. RFS and OS curves were plotted according to the Kaplan-Meier method with significance determined using the log-rank test. All differences were considered statistically significant at the level of *P* < 0.05. Statistical analysis was performed using the IBM Statistical Product and Service Solution, IBM SPSS version 22 (Armonk, New York).

## 3. Results

### 3.1. Calpain-1 Expression in TNBC Tissues

**Figure 1:**
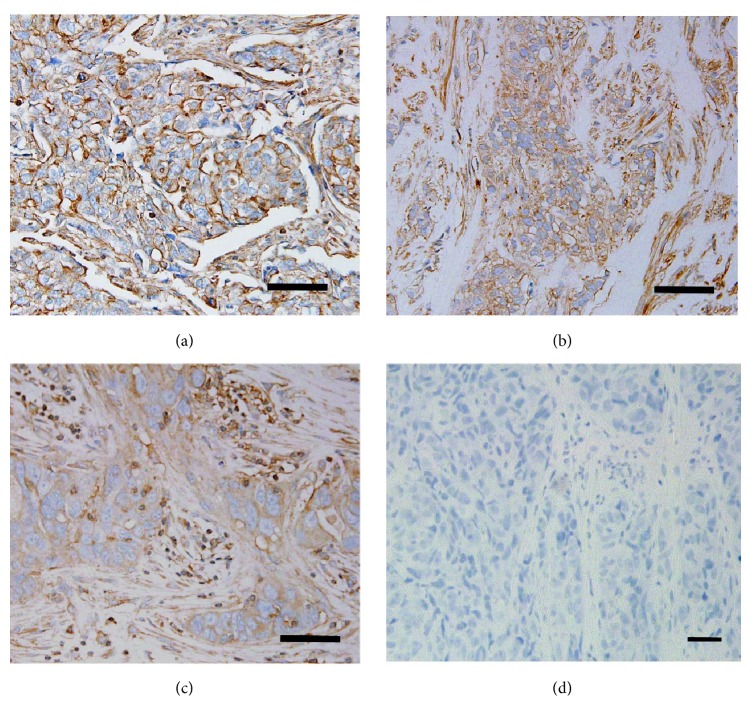
*Detection of calpain-1 protein in TNBC tissues*. Representative immunostaining photomicrographs of high, medium, and low calpain-1 expression [(a) high expression, (b) medium expression, (c) low expression, and (d) negative control (non-TNBC tissue)]. Photomicrographs are shown at ×40 magnification and a scale bar of 20 *μ*m.

Since calpain-1, a Ca^2+^-dependent protease, is mainly found in the cytoplasm [19], therefore, immunohistochemistry was used to determine its location in the TNBC tissues. Immunostained tissues revealed cytoplasmic and membranous staining with some granularity and heterogeneity between adjacent tumor cells, varying from weak to intense staining. Photomicrographs demonstrating representative staining patterns are shown in Figure 1. As shown in Table 1, calpain-1 staining was high in 29.6% (16 of 55) of the cases analysed. It showed intermediate staining in 38.2% (21 of 55) of the cases and low staining in 32.7% (18 of 55). Approximately 96.4% (53 of 55) of the tumors analysed were infiltrative ductal carcinoma, 1.8% (1 of 55) were infiltrative lobular carcinoma, and 1.8% (1 of 55) were micropapillary carcinoma.

### 3.2. Association of Calpain-1 Protein Expression with Clinicopathological Variables

In order to investigate the possibility of using calpain-1 protein as a prognostic biomarker in triple-negative breast cancer, its expression was assessed for association with a number of clinicopathological variables. As shown in Table 2, there was a significant association between calpain-1 expression and the lymph node status (*P* = 0.02) but not with the other clinicopathological variables.

### 3.3. Calpain-1 Expression and Clinical Outcome of TNBC Patients

**Figure 2:**
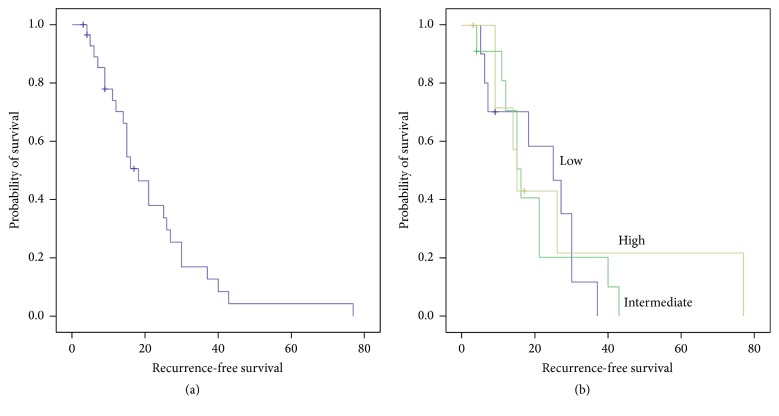
*Kaplan-Meier analysis of TNBC patients' overall survival*. (a) Recurrence-free survival and (b) the impact of high, intermediate, and low calpain-1 protein expression. Calpain-1 expression is not significantly associated with recurrence-free survival (*P* = 0.71) with significance determined using the log-rank test.

**Figure 3:**
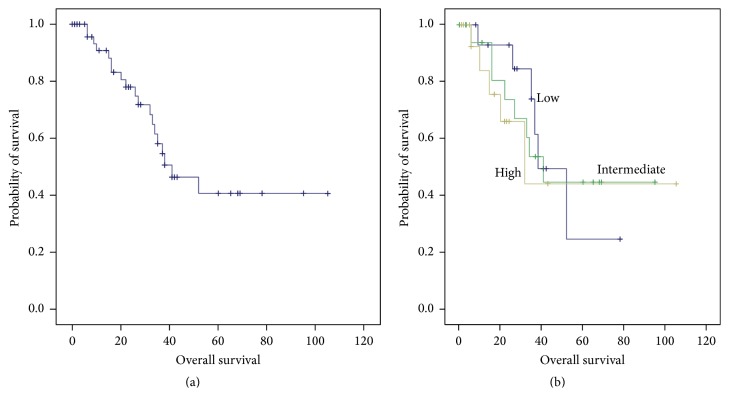
*Kaplan-Meier analysis of TNBC patients' survival and calpain-1 expression*. (a) Overall survival and (b) the impact of high, intermediate, and low calpain-1 protein expression. Calpain-1 expression is insignificantly associated with overall survival (*P* = 0.88) with significance determined using the log-rank test.

To determine the relationship between calpain-1 protein expression in the recurrence-free survival (RFS) and in the overall survival (OS) patients, the Kaplan-Meier survival curves were plotted with significance determined using the log-rank test. The expression of calpain-1 in the triple-negative tissues was not significantly associated with breast cancer RFS (*P* = 0.71, Figure 2) or OS (*P* = 0.88, Figure 3) in which the median RFS was 18 months (3–77 months) and OS was 41 months (0–105 months) in the total patient cohort.

### 3.4. Correlation between Calpain-1 Expression, Cell Proliferation, and Apoptosis in TNBC Tissues

**Figure 4:**
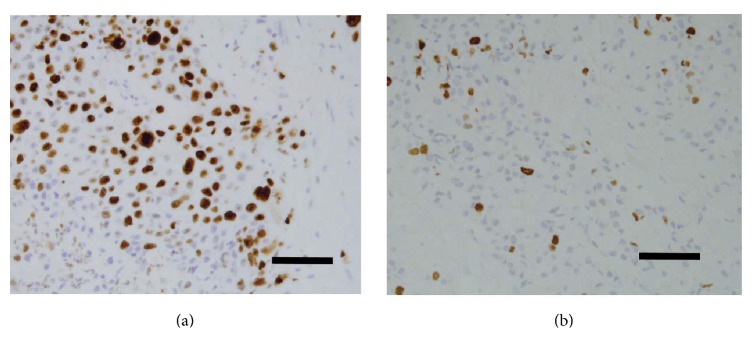
*Immunostaining detection of Ki-67 protein in TNBC tissues*. Cellular proliferative activity of breast cancer tissue was evaluated as high (a) and low (b) percentages using anti-Ki-67 antibody by Immunohistochemistry. Photomicrographs are at ×40 magnification where scale bar shows 20 *μ*m.

**Figure 5:**
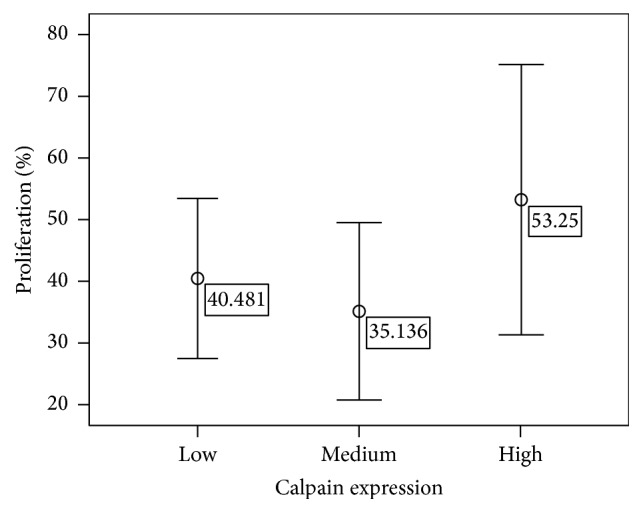
The correlation between calpain-1 expression and proliferation (CI = 95%).

Among the 15 members of the calpain family, the ubiquitous calpain-1 has been implicated in the progression of the cell cycle and in cell proliferation [10]. Ki-67 is considered to be an excellent marker for determining cellular proliferation due to its presence of Ki-67 during all active phases of the cell cycle (G(1), S, G(2), and mitosis) and its absence from resting cells (G(0)) [21]. Cellular proliferative activity of breast cancer tissue was evaluated by IHC staining using Ki-67 antibody, which was estimated as the percentage of tumor cells stained per field ×40 (Figure 4). Statistical analysis showed no significant correlation between calpain-1 expression and proliferation (*P* = 0.29, Figure 5).

**Figure 6:**
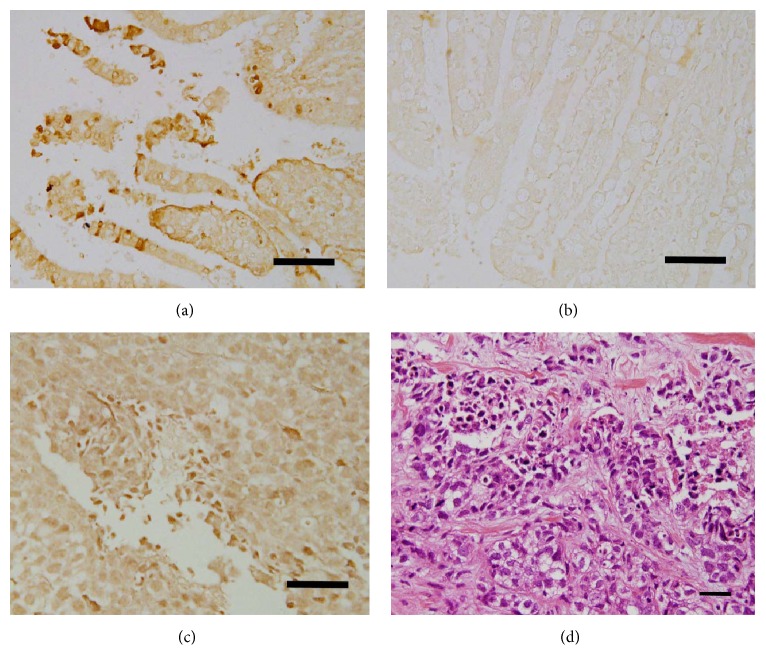
*Evaluation of apoptotic index using H&E and TUNEL staining*. ((a) human colon mucosa, positive control, (b) human colon mucosa, negative control, (c) highly expressed apoptotic cells stained by TUNEL method, triple-negative breast cancer tissue, and (d) apoptotic cells stained with H&E). Photomicrographs are at ×40 magnification and a scale bar of 20 *μ*m.

Apoptosis, in addition to its role of normal development and tissue homeostasis, is also perceived to control the growth of tumor cells by counterbalancing the proliferation rate [22]. Therefore, two approaches: the conventional H&E staining method and the apoptotic TUNEL assay, were both used to detect apoptotic cells and determine whether the frequency of apoptosis was related to tumorigenesis. In addition, the relationship between apoptosis and proliferation was investigated in TNBC tissues. Apoptotic counts using either method, were significantly correlated (*P* < 0.001, *r* = 0.547). Some photomicrographs of TUNEL assay are shown in (Figure 6). Apoptotic bodies were identified and counted according to well-established morphological criteria that included: cell shrinkage, eosinophilic dense cytoplasm with round and smooth margin separating from adjacent cells, chromatin condensation, and nuclear fragmentation (Figure 6(d)). For all of the patients, high apoptotic counts were significantly correlated with increased cell proliferation (*P* = 0.045).

**Figure 7:**
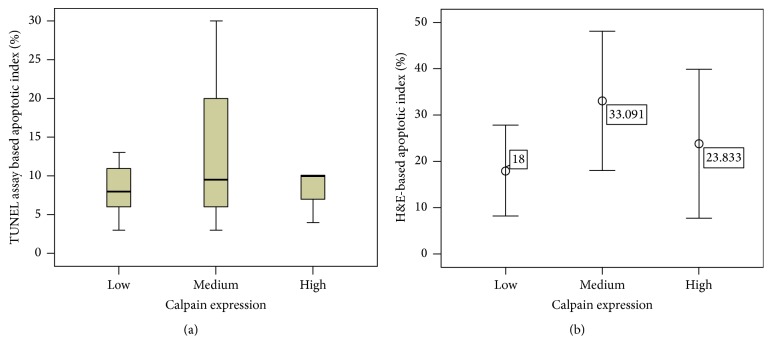
The correlation between calpain-1 expression and TUNEL-based apoptotic ((a) CI = 95%) and H&E-based apoptotic ((b) CI = 95%) staining.

**Figure 8:**
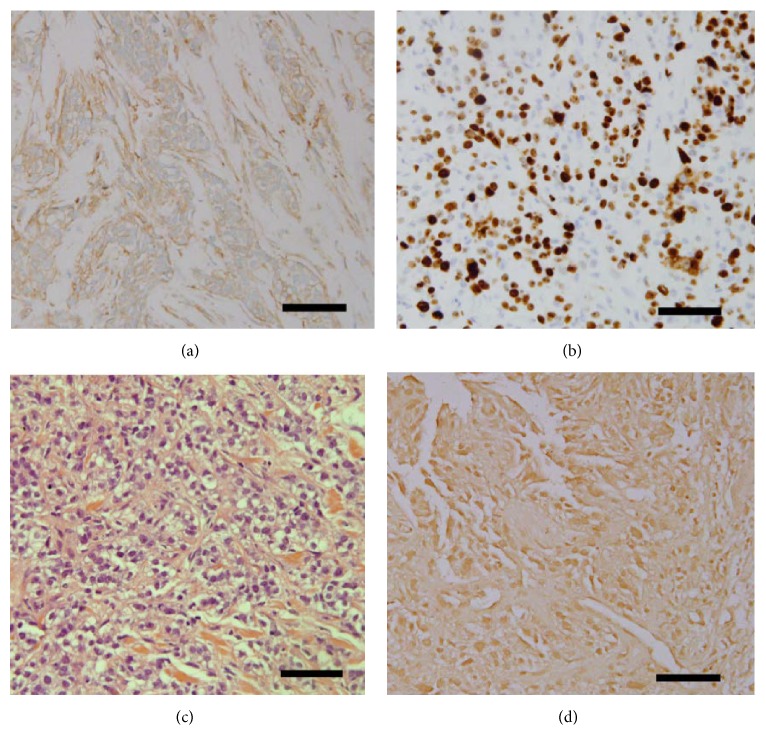
*Representative photomicrographs of series of specimen from the same patient*. (a) Calpain-1 (medium), (b) Ki-67 (70%), (c) apoptosis-based H&E satin (60%), and (d) apoptotic-based TUNEL assay (60%). Photomicrographs are at ×40 magnification and a scale bar equals 20 *μ*m.

The roles for Calpain-1 are paradoxical, in that it is also involved in the regulation of apoptosis [10]. The relationship between calpain-1 expression and apoptosis using the two methods, H&E-based apoptotic counts and apoptotic counts derived from the apoptotic TUNEL assay, was investigated in the TNBC tissues. Interestingly, the data revealed that there was no significant association between neither of them when compared to calpain-1 expression (*P* = 0.710 and *P* = 0.100, Figure 7), respectively. A series of specimens from the same patient is presented in Figure 8 with the evaluation of the result.

## 4. Discussion

Our study was the first to assess the proliferating and apoptotic index in triple-negative breast cancer (TNBC) tissue and to determine its correlation to calpain-1 expression. Such an approach was investigated by measuring the expression levels of calpain-1, ki-67, and apoptosis in TNBC patients using IHC, H&E stain, and TUNEL assay. Moreover, the expression of calpain-1 was assessed for association with a number of clinicopathological variables, clinical outcome, proliferation, and apoptosis. Finally, our study compared between proliferation and apoptotic counts and if there was a correlation between apoptotic counts obtained by H&E staining and TUNEL assay.

The link between calpain system and cancer in general is well-established but yet to be explored specifically in TNBC. It has been demonstrated earlier that calpain activity was significantly higher in breast cancer tissues compared with those of normal breast [23]. Several studies later it was shown that calpain-1 expression was significantly associated with tumor grade [19], proliferation [10, 23–25], and apoptosis [6, 8–11, 25, 26]. Our findings are consistent with these studies in which we showed that calpain-1 was significantly expressed by triple-negative breast cancer tissue at variable intensity ranking from low to high. Moreover, our findings showed significant association with these levels of expression and the lymph node status. Since lymph node status is a tumor-related prognostic factor as stated by the TNM classification [27], our results suggest that calpain-1 might be used as a prognostic factor in TNBC. Calpain-1 was also found to be associated with lymph node status in other types of cancer, such as renal cell carcinoma [28].

Although a strong association was found with the lymph nodes, the lack of association with other clinicopathological variables was consistent with previously published results. One of which is a study conducted by Storr et al. [19] in which they demonstrated a correlation between calpain-1 expression and tumor grade despite the other used clinicopathological variables. Since TNBC is an aggressive form of breast cancer, the tumor grade and/or lymph node status are essential in determining its prognosis. The variations among the presence or absence of association can be explained by certain theories, one of which is that most of patient samples were of intermediate grade tumor and calpain-1 role may have started at later stages as suggested by its correlation with the lymph node status. The lack of wide range of sample collection in regard to tumor grades may have created a diversion in the statistical analysis. In addition to that, the presence or absence of the hormonal receptors such as ER, PR, and HER2 that determine breast cancer behavior and thus treatment can influence the outcome. For example, the expression of calpain-1 has been previously reported in HER2-positive breast cancer patients treated with trastuzumab following adjuvant chemotherapy and no association was found with any of the clinicopathological variables [8, 9].This observation is consistent with our current data but may differ in terms of the positivity of HER2. Another possible theory might be due to the genetic difference between the population in the current study and the published ones, which may affect the expression of calpain-1 in breast cancer cells [19]. Furthermore, the number of samples might have been insufficient to demonstrate any significant correlations.

Normal breast development is regulated by a balance between cell proliferation and apoptosis. This balance plays an essential role in the control of tumor growth. Progression of tumor growth is characterized by increased number of tumor cells, which could be due to increased proliferation and/or decreased apoptosis, or both [29]. Apoptosis is a frequent phenomenon in breast cancer that can be detected by light microscopy in conventional histopathological sections or by special staining techniques. The number of apoptotic cells as a percentage of cells present is known as the AI [20, 30]. Apoptosis is a complex, tightly regulated cellular process that can be divided into three phases: initiation phase, effector phase, and degradation phase. H&E staining is a morphological apoptotic assay that can be subjective and detect apoptosis in its degradation phase, while TUNEL is a molecular-based apoptotic assay that can detect apoptosis in its early phases and is more objective. Therefore, both of them tested apoptosis from different aspects, but the outcome is the same. They were initially used in conjugation to obtain more accurate counts and to prove that the two methods are correlated. There was a significant association between apoptotic scores quantified using H&E in conventional histopathological sections and those quantified using the TUNEL technique which is consistent with a previously published paper (Liu et al., 2001). The highly associated apoptotic counts, obtained using both approaches, indicate the reliability of the generated results. Calpains were notably found to be involved in the apoptosis of breast cancer [10, 26]. The non-TNBC (MCF-7) cells treated with genistein have shown an increase in the intracellular concentration of calcium, leading to the activation of calpain-1, which cleaves and activates caspase-12, hence inducing the apoptosis of the MCF-7 cells [10]. A similar involvement of calpains was also found in the apoptosis of non-TNBC cells triggered by resveratrol and HL-37. Both drugs resulted in an increase in the levels of intracellular calcium followed by an activation of calpains [10]. Our findings, however, indicate the absence of any association between calpain-1 expression and apoptosis in TNBC. These cells may undergo apoptosis through other pathways, for example, via p53-dependent pathway [20, 31] or via other members of the calpain family, such as calpain-2.

Calpains were also found to be involved in the proliferation of breast cancer cells [8, 9, 23, 32, 33]. However, the role of the calpain family in proliferation of TNBC cells has not been reported yet and our preliminary findings show no correlation between calpain-1 expression and proliferation and the same for apoptosis in these cells. Possible theories of the presence and absence of the hormonal receptors, differences in the genetic makeup, and other members of calpains involvement may also influence the correlation with proliferation.

In conclusion, our data clearly demonstrate that there is no correlation between calpain-1 expression and the proliferating/apoptotic index or clinicopathological variables except with the lymph node status of TNBC patients. We also observed that there is a significant correlation between apoptotic counts obtained by both H&E method and TUNEL assay.

Altogether, the significant correlation of calpain-1 expression with lymph node metastasis suggests it as a potential prognostic marker for TNBC; the absence of its correlation with proliferation or apoptosis indicates that it is independent of both processes and its expression might be used as a measure of tumor prognosis.

## Figures and Tables

**Table 1:** Patient's characteristics and association between calpain-1 protein expressions.

	*N* (%)
Females	55 (100)
Median age	
<40 years	17 (30.9)
40–60 years	32 (58.2)
>60 years	6 (10.9)
Menstruation history	
Premenopausal	34 (61.8)
Postmenopausal	21 (38.2)
Family history	
No	45 (81.8)
Yes	6 (10.9)
Not available	4 (7.3)
BMI	
Normal	15 (27.3)
>25–30	14 (25.5)
>30–35	15 (27.3)
>35	8 (14.5)
Missing	3 (5.4)
Breast side	
Left breast	31 (56.4)
Right breast	24 (43.63)
Disease stage	
Stage I	3 (5.5)
Stage II	24 (43.6)
Stage III	21 (38.2)
Stage IV	7 (12.7)
Pathological type	
Infiltrative ductal carcinoma	53 (96.4)
Infiltrative lobular carcinoma	1 (1.8)
Micropapillary carcinoma	1 (1.8)
Ki67 staining	
<20%	9 (16.3)
>20%	45 (81.8)
Missing	1 (1.8)
Calpain protein expression	
Low	18 (32.7)
Intermediate	21 (38.2)
High	16 (29.6)

BMI: body mass index.

**Table 2:** Correlation between calpain-1 protein expression and clinicopathological variables.

Clinicopathological variables	*P* value
Age group	0.512
Menstrual status	0.20
BMI	0.12
Clinical tumor size	0.35
Clinical nodal status	0.02
Clinical stage	0.15
Pathological type	0.33
Pathological tumor size	0.27
Pathological nodal status	0.27
Pathological grade	0.12
Lymphovascular invasion	0.21

BMI: body mass index.

## Abbreviations

DAB: Diaminobenzidine
H&E: Haematoxylin and Eosin
IHC: Immunohistochemistry
ER: Estrogen receptors
HER2: Human epidermal growth factor receptor 2
OS: Overall survival
PBS: Phosphate buffer saline
PgR: Progesterone receptors
PBS: Phosphate buffered saline
RFS: Recurrence-free survival
rTdT: Recombinant Terminal Deoxynucleotidyl Transferase
TUNEL: Terminal deoxynucleotidyl transferase-mediated dUTP nick end labeling
TNBC: Triple-negative breast cancer.
